# From Signal to Certainty: Why COPD Needs More Than One Trial

**DOI:** 10.1007/s00408-026-00914-x

**Published:** 2026-07-31

**Authors:** Mario Cazzola, Daiana Stolz, Don D. Sin, Maria Gabriella Matera, Paola Rogliani

**Affiliations:** 1 Unit of Respiratory Medicine, Department of Experimental Medicine, University of Rome ‘Tor Vergata’, Rome, Italy; 2 Department of Pneumology, University Medical Center Freiburg, University of Freiburg, Freiburg, Germany; 3 Centre for Heart Lung Innovation, Columbia Division of Respiratory Medicine, St. Paul’s Hospital, University of British, Vancouver, BC Canada; 4 Unit of Pharmacology, Department of Experimental Medicine, University of Campania ‘Luigi Vanvitelli’, Naples, Italy

## Abstract

The U.S. Food and Drug Administration’s proposal to adopt a single randomized controlled trial (RCT) as the default evidentiary standard for drug approval marks a substantial change in regulatory philosophy. Although advances in mechanistic science, biomarkers, and statistical methods may justify this approach for conditions with significant, biologically coherent treatment effects, applying it to chronic obstructive pulmonary disease (COPD) raises substantial concerns. COPD is a heterogeneous, multifactorial syndrome with variable disease trajectories, modest treatment effects, and limited validated biomarkers. In this context, reliance on a single trial increases inferential fragility, risks type I error, and limits generalizability due to restrictive eligibility criteria and contextual variability. Although biomarker- and trait-based strategies are promising, they remain insufficiently validated to ensure robust estimation of treatment effects across populations. Similarly, the modest effect sizes and endpoint variability in COPD trials amplify the risk of false-positive or context-specific findings. Replication across independent studies primarily serves to test the consistency and robustness of observed effects under varying conditions, rather than to increase statistical power. We discuss a conceptual regulatory framework in which a single RCT may be acceptable only if it meets strict criteria, including large effect sizes, strong biological plausibility, a low risk of bias, and consistent subgroup effects. However, for highly heterogeneous conditions such as COPD, at least two independent studies remain preferable. Alternatively, if a single robust RCT is conducted, equivalent post-marketing validation is required. Ultimately, regulatory standards should be calibrated to biological and methodological uncertainty, balancing timely patient access with evidentiary reliability.

## Key Points

- The biological heterogeneity of COPD and the modest effects of its treatments make single-trial evidence particularly vulnerable to bias, variability, and limited generalizability.
- Current COPD biomarkers and precision medicine approaches lack sufficient validation to eliminate the need for independent replication.
- Replication across trials provides an empirical safeguard against false positives and context-dependent findings, rather than merely increasing statistical power.
- A flexible regulatory framework should permit single-trial approval only under strict conditions while maintaining multi-trial evidence or imposing strong post-marketing requirements for complex diseases such as COPD.

## Introduction

The U.S. Food and Drug Administration’s (FDA) proposal to adopt a single, randomized, controlled trial (RCT) as the default standard of evidence for marketing authorization represents a substantial shift in regulatory philosophy. The agency claims that advancements in mechanistic sciences, biomarker validation, and Bayesian approaches to trial interpretation may reduce the need for replication traditionally required to control type I error and ensure regulatory certainty [1].

While this reasoning may be compelling in fields with large and consistent treatment effects and well-defined biological targets, such as oncology, applying it to complex, heterogeneous, and nonlinear diseases such as chronic obstructive pulmonary disease (COPD) raises important methodological and clinical concerns. COPD is characterized by multiple overlapping phenotypes, variable disease trajectories, and fluctuating symptom burden. Additionally, exacerbations have multifactorial drivers, including environmental exposures, comorbidities, and infections [2]. Treatment effects are often modest, key endpoints such as exacerbation rates and health status are inherently variable, and validated surrogate biomarkers remain limited [2]. These features introduce substantial uncertainty in the estimation and interpretation of treatment effects within a single-trial framework.

Although the discussion has broader regulatory implications, COPD is used as a model disease to illustrate how evidentiary standards perform in a condition characterized by substantial variability in disease expression, clinical outcomes, and treatment response. In this context, under the current evidentiary conditions in COPD, independent replication through a second adequately powered trial may be a pragmatic safeguard against inferential uncertainty that a single study alone may not fully address.

Evaluating this proposed evidentiary framework requires consideration of several complementary domains, including biological complexity, methodological and statistical challenges, the limitations of current precision medicine approaches, questions of external validity, and broader regulatory and policy implications.

## Biological Complexity and Inferential Fragility

COPD exhibits significant biological and clinical heterogeneity, reflecting a range of partially overlapping phenotypes and potential endotypes. This variability encompasses symptom severity, lung function, susceptibility to exacerbations, inflammatory markers, and the impact of comorbid conditions [3].

In this context, relying on a single large trial, even if it is methodologically rigorous, can amplify inferential fragility and increase the risk of type I error, especially when multiple interrelated hypotheses are implicitly assessed within a unified mechanistic framework [4]. Regulatory science has long addressed these risks through prespecified multiplicity control procedures, including hierarchical testing strategies and alpha-spending approaches, which are designed to maintain an acceptable family-wise error rate in confirmatory evaluations [5].

The FDA’s 2026 Draft Guidance on Substantial Evidence addresses this issue by emphasizing that evidentiary strength should not be reduced to a binary interpretation of statistical significance [6]. Instead, the guidance encourages a multidimensional appraisal that integrates the observed p-value, prior probability of benefit, Bayesian posterior estimates, and the clinical importance and magnitude of the treatment effect. Within this framework, statistically significant findings from large studies may still correspond to limited clinical relevance, while more modest statistical signals can be meaningful when they affect outcomes such as mortality or irreversible functional decline. The document also notes that the conventional one-sided significance level of 0.025 may not always adequately control for false positive conclusions when the prior probability of efficacy is low. Alternative thresholds may be justified when supported by robust prior evidence or corroborating data from independent sources. Together, these perspectives support shifting from a rigid threshold-based interpretation to a more integrated assessment of evidentiary credibility.

A further challenge concerns external validity. Eligibility criteria in COPD trials are often restrictive, resulting in study populations that represent only a narrow and relatively stable subset of patients. This limits applicability to routine clinical practice [7, 8].

Replication across two studies with comparable inclusion criteria does not fully overcome this limitation, but it partially strengthens the evidentiary base by enabling comparison across independent experimental contexts [9]. When conducted in different geographical regions and healthcare systems, trials inevitably incorporate variation in patient characteristics, clinical pathways, environmental exposures, and standard-of-care practices [10]. While these differences do not eliminate selection bias, they offer a practical way to test robustness that a single trial cannot provide [9].

Furthermore, even with similar protocols, differences between trials in adherence patterns, background therapies, exacerbation definitions, and healthcare delivery may influence the observed treatment effects and reveal inconsistencies that would otherwise remain undetected in a single study [11]. Thus, replication primarily functions as a test of stability across heterogeneous research environments rather than as a mechanism to increase patient diversity per se [12]. Finally, while pooled analyses of trials conducted with identical designs can improve precision and reduce uncertainty around effect estimates [13], they do not substitute for independent replication because they cannot evaluate reproducibility across distinct clinical and operational contexts.

## Limitations of Current Biomarker and Trait-Based Strategies

The FDA’s position that mechanistic coherence and validated surrogate endpoints justify approval based on a single pivotal RCT [1] is difficult to reconcile with current COPD practice. In COPD, diagnosis and management still rely primarily on clinical indices rather than direct measures of underlying pathobiology. Most candidate biomarkers remain insufficiently validated for regulatory use [3, 17].

Biomarker-driven and treatable-trait strategies are an important step forward [14, 15], yet enrichment strategies do not automatically produce more robust evidence. Instead, they may intensify the trade-off between individualized selection and the need for broadly representative studies capable of capturing treatment effects across different populations [16].

Although biomarkers have many uses, including diagnosis, prognosis, monitoring, pharmacodynamic assessment, and evaluation of surrogate endpoints [17], we will limit our discussion to their use in predicting treatment response before therapy initiation. This predictive application differs conceptually from a post hoc assessment of response, and each requires a different validation framework and evidentiary standard [18].

Of the available markers, blood eosinophil count is the most extensively validated in COPD [16], and the Global Initiative for Chronic Obstructive Lung Disease (GOLD) recommends its use to predict the effect of inhaled corticosteroids (ICS) on exacerbation prevention [2]. The relationship with treatment benefit appears to be continuous, with minimal effect around 100 cells/µL and progressively greater benefit at higher thresholds, particularly ≥ 300 cells/µL. However, its utility is constrained by several important limitations: proposed cut-offs vary across trials (100–300 cells/µL), variability increases at higher levels, and predictive performance is influenced by exacerbation history, ICS use, smoking status, ethnicity, and geography. Reproducibility is moderate to high (intraclass correlation coefficient: 0.64–0.89), but concordance between blood and airway eosinophils is inconsistent (r: 0.18–0.70), reflecting sampling variability and differences in tissue distribution [19]. Importantly, GOLD does not currently support its use in predicting exacerbation risk at the individual level [2]. For other proposed biomarkers, such as those reflecting dysbiosis, persistent systemic inflammation, or impaired repair mechanisms, prospective validation for treatment stratification remains limited or inconsistent [19, 20].

In this context, replication across independent trials is a way to test whether biomarker-defined effects remain stable across populations. This strengthens the credibility of subgroups and reduces the likelihood that observed associations arise from unstable thresholds or model overfitting [21, 22]. More broadly, the limited reproducibility and variable predictive performance of existing COPD biomarkers underscore the need for confirmatory evidence instead of relying on single enriched datasets [17, 23, 24].

Ideally, mechanistic in vivo studies would corroborate target engagement and pathway modulation [25]. However, regulatory decisions are often made in the absence of fully elucidated mechanisms. Whether an endpoint reasonably predicts clinical benefit depends on biological plausibility and supporting empirical evidence, which is assessed on a case-by-case basis [26]. We are not arguing that such evidence should be mandatory. Rather, when available, it may compensate for reduced replication by strengthening causal inference, analogous to situations in which the FDA accepts a single pivotal RCT supported by related evidence [27]. In its absence, the role of independent replication becomes more important, aligning with the historical standard established by the 1962 statutory requirement of two adequate and well-controlled studies to reduce the risk of undetected bias in single trials [25].

Experience with targeted biologic therapies illustrates both the potential and limitations of biomarker-guided development. For example, trials of mepolizumab in COPD have shown that it is difficult to consistently identify responsive subgroups, even when using eosinophilic enrichment strategies [28]. In contrast, the BOREAS and NOTUS trials of dupilumab showed consistent reductions in exacerbations among patients with type 2 inflammatory signatures [29], suggesting that replication is more feasible when biologically coherent endotypes and biomarker profiles are used to guide patient selection.

## Effect Size, Signal Detection, and Statistical Considerations

A single-trial regulatory paradigm may unintentionally favor settings in which the treatment signal is strong relative to background variability, while insufficiently capturing a complex syndrome with substantial divergences in clinical presentation, physiology, and response to therapy. One example is COPD [2]. The required sample sizes and follow-up durations to demonstrate disease modification in COPD depend on the endpoint and methodology employed. For example, when assessing disease progression using longitudinal lung function decline, studies have estimated that approximately 1,000 patients per group followed for three years are needed to detect a 50% reduction in disease progression with adequate statistical power [30]. A single-trial framework may be more appropriate in cases where treatment effects are large, rapid, and mechanistically well defined, particularly when interventions target biologically coherent pathways or highly selected patient populations. In such settings, effect sizes may exceed background variability, thereby reducing inferential uncertainty and strengthening the robustness of evidence derived from a single study.

Conducting two trials does not improve signal detection by itself, and averaging results across studies can obscure true effects. Nevertheless, replication increases confidence that positive findings reflect genuine treatment effects, even when underlying probabilities remain low [31]. The primary purpose of replication is not to enhance statistical power, but rather to reduce the probability that statistically significant findings arise from random variation, bias, or context-specific influences [27].

In COPD trials, the effect sizes for reducing exacerbation frequency are generally modest and remain a matter of debate [32]. Since exacerbations are discrete clinical events rather than continuous patient-reported outcomes, the concept of a minimal clinically important difference (MCID) is not directly applicable, Consequently, no validated MCID has been established for COPD exacerbation frequency [32]. As a result, the discussion has focused on clinically relevant effect sizes. Available evidence suggests that reductions in exacerbation frequency of approximately 11% to 20% are generally considered clinically meaningful [33, 34]. Recent large COPD trials have been powered to detect a 15% reduction in exacerbation frequency. This threshold was selected following consultation with clinicians who considered it a small but clinically important treatment effect [33]. This contrasts with oncology, where large and rapid effects on validated surrogate endpoints substantially reduce inferential uncertainty [35].

In settings with modest effect sizes, the risk of false positive and false negative findings increases because of multiplicity, endpoint variability, or subgroup instability. As the number of comparisons increases, so does the probability of obtaining a statistically significant result by chance [36]. Independent replication is an important safeguard against these risks and offers greater assurance that the observed effects are reproducible and not artefacts of a specific study context [27].

### Reconsidering the Oncology Paradigm

The FDA cites oncology as a setting in which limited pre-approval evidence is sufficient [1]. In these cases, the substantial strengthening of evidentiary confidence and the appropriateness of expedited regulatory pathways were due to the large and rapid effects on validated surrogate endpoints combined with a well-defined molecular mechanism of action [37]. Despite a limited investigational base, the magnitude, consistency, and biological plausibility of effects supported approval.

Single-trial approvals are less consistent outside of oncology, generally relying on effect sizes, clear physiological mechanisms, or urgent unmet needs. Regulatory pathways such as Fast Track recognize that single trials can be sufficient for therapies addressing unmet needs or serious or life-threatening conditions [38]. However, these criteria are rarely fulfilled in COPD, where drug development is hindered by incomplete understanding of disease biology, limited in vitro and in vivo models, poorly validated biomarkers, and inefficient endpoints. Consequently, COPD has the lowest estimated probability (16%) of a drug reaching the market among the 11 major disease areas studied [30].

### Do Two Trials Change Decisions?

Empirical analyses across therapeutic areas suggest that second pivotal trials often fail to replicate initial positive findings or yield smaller effect sizes [39], underscoring the need for confirmatory evidence. However, only 19.5% of 185 single pivotal trial approvals by the FDA from 2015 to 2023 referred to supporting evidence [40], suggesting an inconsistent application of the more rigorous assessment criteria.

There is limited COPD-specific data, but broader evidence suggests that replication can affect conclusions on efficacy and robustness. A recent example is the Phase 3 clinical program of itepekimab, a fully human monoclonal antibody targeting interleukin-33, a cytokine involved in epithelial-driven inflammatory pathways relevant to COPD. The program included two large, parallel, pivotal studies (AERIFY-1 and AERIFY-2) in patients with moderate-to-severe disease. In AERIFY-1, itepekimab reduced moderate-to-severe exacerbation rates by 27% versus placebo, suggesting potential benefit. However, AERIFY-2 failed to replicate these findings, showing no significant treatment effect over approximately one year of follow-up [41].

The discordant findings of the AERIFY program illustrate the type of inconsistency that some regulatory authorities may interpret as evidence requiring further confirmation. Notably, the European Medicines Agency (EMA) has historically required at least two positive, adequate, and well-controlled confirmatory trials as the evidentiary standard for approval, reflecting a philosophy that prioritizes reproducibility and consistency over isolated statistically significant findings [42].

## Generalizability and Trial Populations

Many pivotal COPD trials differ more in their geographic distribution than in their enrolled populations, reflecting significant differences in exacerbation rates across countries [43]. These differences limit generalizability due to differences in environmental exposures, healthcare access, and clinical practice patterns [44]. In fact, only 17%-42% of primary care patients with COPD in the real world would qualify for major industry-sponsored trials, which typically enroll younger patients, predominantly men, with more severe lung function impairment [45, 46].

Temporal variability may also influence trial outcomes. For example, the SARS-CoV-2 pandemic substantially altered respiratory infection patterns, healthcare utilization, exacerbation rates, recruitment, and study conduct in COPD trials [47]. These disruptions demonstrate that treatment effects observed during a specific period may not always be generalizable to other settings or timeframes, highlighting the importance of confirming findings across different circumstances.

Future trial programs should prioritize patient diversity by minimizing unnecessary exclusions, building community partnerships, and adapting interventions to patients’ needs [48]. Incorporating this approach into future studies may enhance participation among underrepresented groups, yielding results that better reflect patients with a disproportionate burden of the disease.

## Regulatory Considerations and Future Directions

**Fig. 1:**
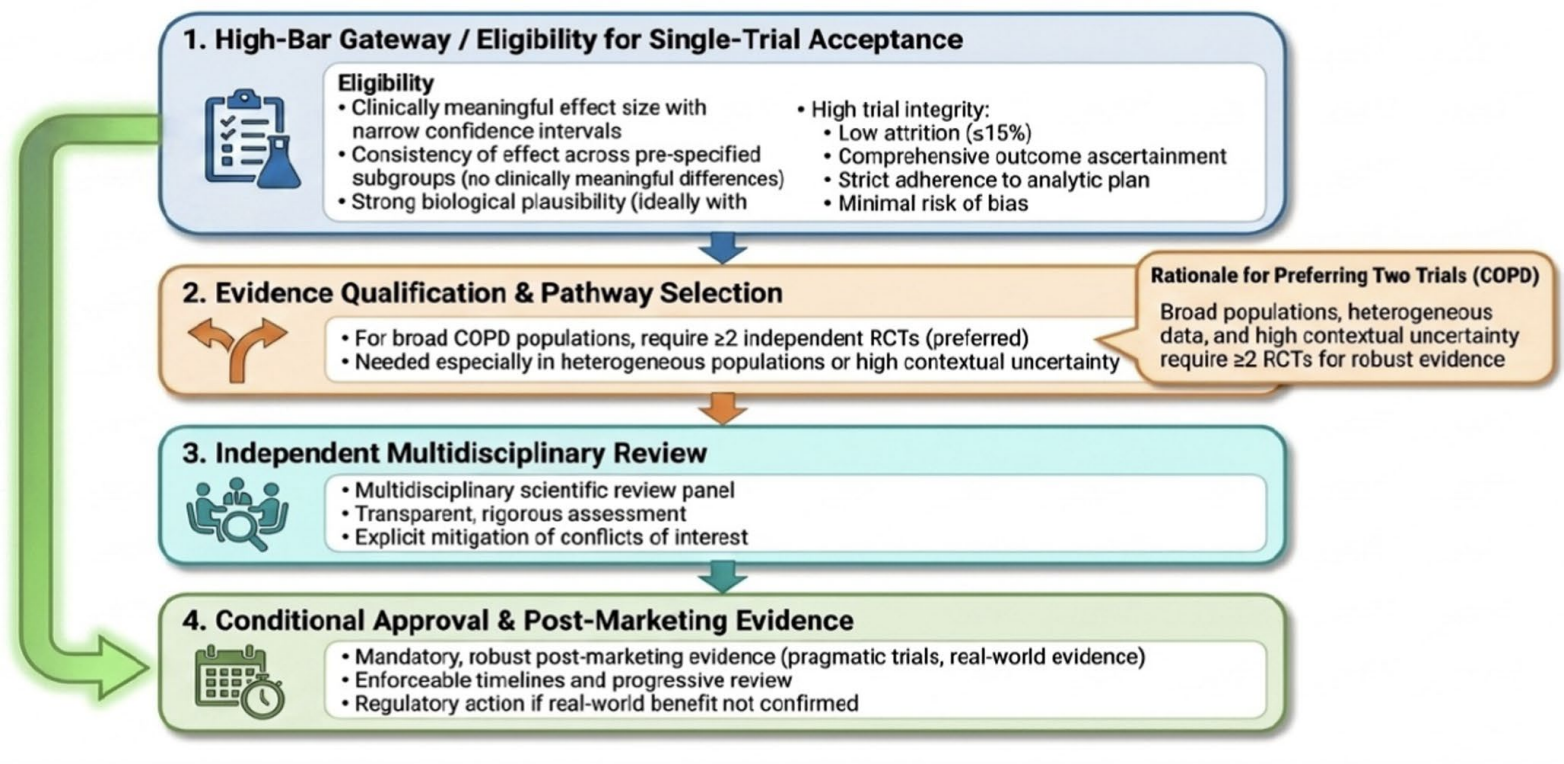
Proposed framework for combining different types of evidence in developing drugs for COPD. Conceptual representation of how confidence in treatment effects in COPD can be enhanced by integrating multiple sources of evidence, including randomized trials, replication studies, real-world data, and biological plausibility. This framework reflects the authors’ perspective and is presented for discussion purposes

To improve clarity and practicality, we present a conceptual framework for evaluating innovative strategies for generating scientific data in the clinical development of COPD (Fig. 1). The framework comprises four elements: (1) a high-bar gateway for single-trial acceptance, (2) evidence qualification and regulatory pathway selection, (3) independent multidisciplinary review, and (4) conditional approval supported by robust post-marketing evidence generation. Rather than proposing a COPD-specific regulatory model, this framework is intended to stimulate discussion on applying existing regulatory principles when conventional evidentiary standards are difficult to achieve because of disease heterogeneity, modest treatment effects, and challenges in endpoint assessment.

Within the proposed framework, reliance on a single RCT may be acceptable if the following stringent, predefined criteria are met [49], including: (1) an effect size that is clinically meaningful and accompanied by narrow confidence intervals; (2) consistency of the effect across pre-specified subgroups with no clinically meaningful dissimilarities identified through formal interaction testing [50]; (3) strong biological plausibility, ideally supported by mechanistic evidence of target engagement [40]; and (4) high trial integrity with a minimal risk of bias, including low attrition (≤ 15%), comprehensive outcome ascertainment, and strict adherence to pre-specified analytic plans [51]. Under these conditions, high internal consistency may partially compensate for the absence of independent confirmatory evidence.

The FDA’s regulatory practice has progressively shifted toward greater acceptance of single pivotal trials. Approvals supported by at least two pivotal studies declined from 81% (1995–1997) to 53% (2015–2017) [38]. A similar trend has been observed within the European regulatory framework, where 45% of new active substances approved between 2012 and 2016 were supported by a single pivotal clinical trial [42].

However, this regulatory evolution cannot be directly extrapolated to COPD. Due to the substantial clinical and biological complexity of the disease and the frequent absence of molecular or phenotypic enrichment strategies, independent confirmation remains particularly important. Under these circumstances, robust conclusions are more likely to emerge from evidence generated across at least two adequately conducted RCTs. This perspective aligns with the EMA’s more conservative approach, in which two positive, well-controlled clinical trials continue to serve as the reference standard. EMA assessments typically emphasize confirmatory evidence and consistency across studies, especially for heterogeneous diseases. For example, the traditional two-trial standard was met in only about half of oncology drug approvals between 2014 and 2019 [52]. 

The “totality of evidence” approach used by the EMA integrates multiple sources of evidentiary support, including biological plausibility, consistency across studies, endpoint concordance, supportive analyses, and external evidence, rather than relying on any single evidentiary element in isolation [42]. Replication should be viewed as one component of the totality of evidence rather than an alternative to it. Confirmatory evidence is therefore not merely a formal requirement but one of the most effective means of assessing reproducibility across independent settings, particularly when mechanistic understanding and validated surrogate endpoints are limited.

This consideration is particularly relevant in COPD, where heterogeneity in patient populations, disease expression, and healthcare systems across Europe may complicate the interpretation and generalizability of findings derived from a single pivotal study [53]. Consequently, independent confirmation strengthens the overall evidentiary framework by increasing confidence in observed treatment effects and complementing other sources of evidence within the totality-of-evidence paradigm.

An independent and impartial review is not intended to replace existing regulatory authorities, but rather to strengthen the current evaluation processes by providing transparent, multidisciplinary expertise and reducing susceptibility to potential conflicts of interest. This is particularly important when the evidence is marginal, complex, or derived from a single pivotal trial [54].

When approval is based on a single trial, it should be conditional upon clearly defined, enforceable post-marketing requirements. These requirements should include pragmatic clinical trials and real-world effectiveness studies conducted within specified timelines [45, 55], with meaningful regulatory consequences for noncompliance [25].

The current track record for completing post-marketing studies is suboptimal. Of the drugs granted accelerated approval between 2009 and 2013, only half of the required confirmatory studies were completed within three years [56]. Of the 614 post-approval obligations issued from 2009 to 2010, only 54% were completed by 2015, while 20% had not yet begun, and 25% were still ongoing or delayed [25]. Overall, post-marketing commitments and formal requirements have a completion rate of two-thirds [57]. Therefore, regulatory authorities should retain the authority to restrict or withdraw approval when confirmatory evidence is not generated [55]. Although the FDA’s Food and Drug Omnibus Reform Act of 2022 strengthened enforcement authority [58], effective implementation remains a critical challenge.

## Ethical, Economic, and Innovation Considerations

The FDA initiative is partially justified by economic considerations, such as reduced drug development costs and accelerated market access [59]. However, these potential benefits must be weighed against the risks associated with a lower evidentiary threshold [25]. Earlier approval of effective therapies can meaningfully benefit patients and support pharmaceutical innovation [60]. Conversely, prematurely approving marginally effective therapies can lead to the inefficient allocation of limited healthcare resources and expose patients to treatments that ultimately prove ineffective [61]. Recent examples illustrate this concern. Aducanumab received accelerated approval for Alzheimer’s disease in 2021 [62] despite controversial efficacy data but was voluntarily withdrawn in 2024 following termination of the confirmatory trial [63]. Similarly, sodium phenylbutyrate/ursodesoxycholic acid, approved for amyotrophic lateral sclerosis in 2022, was withdrawn in 2024 after a Phase 3 trial showed no benefit in terms of function, survival, or quality of life compared to placebo [64]. These cases demonstrate that the premature approval based on limited evidence can expose patients to ineffective therapies, waste resources, and undermine regulatory trust. Therefore, the key challenge is not cost reduction itself but rather optimizing the timing of evidence generation to balance early access with evidentiary certainty [65].

### Can Two Trials Hinder Innovation?

Requiring two trials could substantially increase development costs and timelines, which could reduce incentives to invest in COPD therapies. Pivotal trials have a median cost of about $19 million (with an interquartile range of $12-$33 million), although costs vary considerably according to study design [66]. Large trials involving more than 1,000 participants may cost up to $77.2 million, compared to $5.9 million for enrolling 100 patients or fewer. Phase 3 trials are particularly resource-intensive, with a mean enrollment of approximately 630 patients, and they are substantially longer in duration than Phase 1 studies (38 versus 27.8 months) [67]. Requiring two such trials could therefore nearly double overall development costs and prolong time to approval.

This issue is particularly important for smaller companies and therapies that target narrowly defined patient populations. The FDA acknowledges these constraints and accepts a single pivotal trial when conducting a second study is infeasible (e.g., rare diseases) or unethical (e.g., when a clear survival benefit has already been demonstrated) [38]. Therefore, a flexible evidentiary framework may be preferable to a rigid two-trial requirement, provided that reduced pre-approval evidence is offset by robust post-marketing commitments. However, the effectiveness of this approach depends on timely completion and rigorous enforcement of confirmatory studies.

Although the Accelerated Approval pathway requires post-approval confirmatory studies to verify clinical benefit, regulatory oversight and enforcement of these obligations have frequently been inadequate [20]. Among accelerated approvals from 2009 to 2013, eight indications still lacked confirmed clinical benefit five or more years after approval [55]. More recent analyses indicate that over half of the confirmatory trials that were due by 2021 failed to meet their deadlines, and many remain unfinished [68]. When approval is granted based on limited pre-approval evidence, strong regulatory oversight after approval becomes essential [69, 70]. This includes the ability to withdraw approval, impose restrictions, or require additional studies if the expected clinical benefit is not confirmed.

### Drugs vs Devices

Historically, the FDA approved COPD-related medical devices based on a single adequate and well-controlled clinical investigation, often complemented by long-term follow-up or registry data [71, 72]. This approach reflects the more flexible evidentiary framework applied to devices, for which clinical trial data are only required when appropriate, and they may include controlled studies, partially controlled investigations, or well-documented case series [25]. For many devices, large blinded RCTs are considered impractical or unnecessary because clinical performance is also supported by bench testing, animal studies, and engineering evidence [73].

Consequently, concerns about a mandatory two-trial requirement primarily apply to pharmacological therapies, where systemic exposure, long-term treatment, and population-level considerations generally justify more stringent evidentiary standards. Nevertheless, when substantial uncertainty remains, similar principles of rigorous validation and post-marketing evaluation should apply to devices. Although the FDA required at least one post-approval study for 67.9% of high-risk devices, only 18.2% of these studies were reported as completed [71]. This discrepancy underscores the need to enhance oversight of post-marketing commitments, particularly when devices are approved based on limited premarket evidence.

## Conclusion

The FDA’s shift toward a single-trial default contrasts with the EMA’s more continuity-oriented approach (Table 1). While the 1962 statute generally required two adequate and well-controlled RCTs, reflecting concern that single trials may be biased, U.S. legislation in 1997 formalized acceptance of a single pivotal trial under specific conditions [25]. Both agencies retain flexibility for cases of high unmet need, large effect sizes, or strong mechanistic support. However, the EMA places greater emphasis on confirmatory evidence through replication or converging data [74, 75].

This distinction is especially relevant in COPD, where heterogeneity, biological variability, and limited representativeness of trial populations increase uncertainty. Despite having clinically relevant disease, more than half of patients with COPD are excluded from major RCTs [46, 76]. The EMA’s “totality of evidence” approach and post-authorization requirements [42, 77] therefore address uncertainty by ensuring cross-study consistency.

Overall, the FDA and EMA approaches are complementary. Consistent with the proposed framework, a single RCT is only acceptable under the following conditions: strong internal validity, large and clinically meaningful effects, narrow confidence intervals, and low risk of bias. Otherwise, replication or stronger post-marketing evidence is required [49–51]. The FDA emphasizes earlier access under bounded uncertainty, while the EMA reduces uncertainty progressively through replication and post-authorization verification.

The core issue is not single versus multiple trials, but rather, matching evidentiary standards to uncertainty. In COPD, replication or robust post-marketing validation is generally necessary due to modest effect sizes and population heterogeneity. However, highly deterministic settings may justify a single high-quality trial.

**Table 1:** Comparative regulatory approaches of the Food and Drug Administration (FDA) and European Medicines Agency (EMA)

Dimension	FDA	EMA
Primary evidentiary standard	Increasing acceptance of a single pivotal RCT under defined conditions	Traditionally anchored in two independent adequate and well-controlled RCTs, with case-by-case flexibility
Regulatory philosophy	Flexibility-oriented framework, prioritizing timely access and innovation	Continuity- and robustness-oriented framework, prioritizing evidentiary consolidation
Role of replication	Considered supportive but not systematically required	Considered a core mechanism to reduce inferential uncertainty
Use of mechanistic and biomarker evidence	May substitute partially for replication when strongly validated	Considered supportive but insufficient to replace confirmatory clinical evidence
Approach to uncertainty	Greater tolerance of pre-approval uncertainty, offset by post-marketing requirements	Lower tolerance of residual uncertainty at approval stage, mitigated through multiple sources of confirmatory evidence
Handling of heterogeneous diseases (e.g., COPD)	Higher reliance on single-trial evidence, with increased dependence on post-marketing validation	Preference for cross-trial consistency and external robustness across populations and settings
Totality of evidence framework	Integrates clinical and supportive evidence, potentially including single pivotal studies	Explicit multi-study “totality of evidence” approach, emphasizing convergence across datasets
Post-marketing obligations	Required but historically subject to variable completion and enforcement challenges	More systematically integrated into authorization (e.g., conditional approvals with enforceable obligations)
Regulatory flexibility	Applied through expedited pathways (e.g., unmet need, accelerated approval)	Applied through mechanisms such as conditional marketing authorization with structured follow-up
Overall orientation	Earlier access under acceptable uncertainty	Higher pre-approval evidentiary certainty through replication and consistency

## Data Availability

No datasets were generated or analysed during the current study.
